# Supplementary material for: Association between Health-Related Physical Fitness and Self-Rated Risk of Depression in Adolescents: Dados Study
Source: Int J Environ Res Public Health. 2020 Jun 17;17(12):4316. doi: 10.3390/ijerph17124316 (PMC7345278; doi:10.3390/ijerph17124316)
Supplement: Supplementary file 1 [file ijerph-17-04316-s001.pdf]

Table S1. Partial correlations analyses of physical fitness components and risk of depression score.

		Cardiorespiratory Fitness	Upper Limb Muscular Strength	Lower Limb Muscular Strength	Speed-Agility	Body Mass Index	Waist Circumference	Body Fat
Risk of depression	β	−0.159	0.004	−0.014	−0.088	0.141	0.133	0.110
	P	**0.018**	0.957	0.841	0.191	**0.036**	**0.049**	0.102

Statistically significant values are in bold.
